# Prevalence of progressive supranuclear palsy in Yonago: change throughout a decade

**DOI:** 10.1002/brb3.557

**Published:** 2016-10-11

**Authors:** Hiroshi Takigawa, Michio Kitayama, Kenji Wada‐Isoe, Hisanori Kowa, Kenji Nakashima

**Affiliations:** ^1^ Division of NeurologyDepartment of Brain and NeurosciencesFaculty of MedicineTottori UniversityTottoriJapan

**Keywords:** epidemiology, PSP‐parkinsonism, PSP‐pure akinesia with gait freezing, Richardson's syndrome, tauopathy

## Abstract

**Background:**

Progressive supranuclear palsy (PSP) is a neurodegenerative disorder that is sometimes confused with Parkinson's disease, multiple system atrophy, and other disorders. The typical clinical features are categorized as Richardson's syndrome (RS), but other clinical subtypes include PSP‐parkinsonism (PSP‐P) and PSP‐pure akinesia with gait freezing (PSP‐PAGF). In this study, we determined the prevalence of PSP in a Japanese rural area compared to our previous 1999 report.

**Methods:**

We collected data in Yonago City from 2009 to 2014 using a service‐based study of PSP. We collected case history data from PSP patients in the area from our hospital. The crude prevalence and 95% confidence interval (CI) were calculated using the population demographics on the prevalence day of 1 October 2010. Age‐ and sex‐adjusted prevalence was calculated by direct standardization to the population demographics in Yonago City on the prevalence day of 1 April 1999.

**Material and Results:**

We identified 25 patients: 16 with probable RS, 4 with possible RS, 3 with clinical PSP‐P, and 2 with clinical PSP‐PAGF. The prevalence per 100,000 was 17.90 (male = 18.05; female = 17.76). The prevalence of PSP in Yonago in 2010 increased compared to the measurements from 1999.

**Conclusion:**

The prevalence of PSP in Japan increased from 1999 to 2010.

## 1 Introduction

Progressive supranuclear palsy (PSP) was described in 1964 as a distinct neurodegenerative disorder with symptoms of supranuclear ophthalmoplegia mainly affecting vertical gaze, pseudobulbar palsy, and dystonic rigidity of the neck and upper trunk (Steele, Richardson, & Olszewski, 1964). Cytoskeletal tau aggregates are the pathophysiological hallmark of PSP (Dickson, Ahmed, Algom, Tsuboi, & Josephs, 2010; Perez et al., 1998). The typical phenotype of PSP (Richardson's syndrome [RS]) is clinically characterized by vertical gaze palsy or hypometric vertical saccades and postural instability, with falls occurring in the first year, in combination with predominantly axial rigidity, dysarthria, dysphagia, and frontal behavioral abnormalities (Burn & Lees, 2002; Williams & Lees, 2009). Recently, clinical subtypes, such as PSP‐parkinsonism (PSP‐P) and PSP‐pure akinesia with gait freezing (PSP‐PAGF), have been described indicating that the various symptoms have the same pathophysiological background (Williams, Holton, Strand, Revesz, & Lees, 2007; Williams et al., 2005).

Few epidemiological studies have been performed regarding PSP. In 1999, we reported the prevalence of PSP in Yonago City, the western area of the Tottori Prefecture in Japan (Kawashima, Miyake, Kusumi, Adachi, & Nakashima, 2004). In the previous survey, PSP was conceptually equivalent to RS. The subjects were the patients with only RS according to the diagnostic criteria of the National Institute of Neurological Disorders and Stroke and Society for PSP (NINDS‐SPSP; Litvan et al., 1996). The elderly portion of the population of Yonago City is presently increasivng. Accordingly, changes in the prevalence of PSP are predicted. Since 2003, PSP has been listed on the Specified Disease Treatment Research Program of the Japanese government. Moreover, subtypes of PSP have been described during the decade since our previous investigation. In this study, we determined the prevalence of PSP, including subtypes, in a Japanese rural area, Yonago City, and compared these data to our previous report in 1999.

## 2 Patients and Methods

### 2.1 Area of investigation

The investigated area, Yonago City, is located in western Japan (35 degrees north and 133 degrees east). The adjacent Yodoe Town (area, 25.74 km^2^; population, approximately 9,000) became part of the new Yonago City as a result of a municipal merger on 31 March 2005. The new Yonago City covers an area of 132.21 km^2^, and the population was 148,271 (70,133 males and 78,138 females) on 1 October 2010. The area of the initial Yonago City was 106.47 km^2^, and the population was 139,683 (66,483 males and 73,200 females) on 1 October 2010. The population increased from 137,420 in 1999. The percentage of individuals 65 years and over increased from 18.3% in 1999 to 23.4% in 2010. The percentage of individuals less than 15 years old decreased from 16.0% in 1999 to 14.5% in 2010. The population demographics of the Yonago area were received from the Yonago city office. In new Yonago City, there are three general hospitals, five rehabilitation hospitals, and one University Hospital with 33 board‐certified neurologists. Most neurologists in this rural town received their training as a neurologist at the University Hospital and continue to work closely with the University Hospital, as such, the neurological medical services in the area are quite efficient. Typical cases of Parkinson's disease or Alzheimer's disease were occasionally diagnosed at the general hospital. However, most atypical cases or severe cases with parkinsonism or motor symptom patients such as PSP, multiple system atrophy, corticobasal degeneration and amyotrophic lateral sclerosis were referred to the University Hospital, where they underwent detailed examination and diagnosis. The subjects living in the area of former Yonago City were analyzed according to the population demographics generated from the former Yonago City in order to use the same area for comparison with the prevalence of PSP in 1999.

### 2.2 Service‐based study in Yonago City

The survey was performed six times over 6 years from 2009 to 2014. In each survey, case data were collected from the medical records of PSP, Parkinson's disease, corticobasal degeneration, multiple system atrophy, and Parkinson's syndrome patients at the University Hospital.

### 2.3 Diagnosis

The patients participated in an interview regarding their clinical information, including symptoms, clinical course, past history, family history, a detailed neurological examination by board‐certified neurologists, and more examinations (including a magnetic resonance imaging study and 123‐I‐meta‐iodobenzylguanidine myocardial scintigraphy in the Division of Neurology at Tottori University Hospital). RS was diagnosed according to the criteria of the NINDS‐SPSP. We researched in detail the clinical history of the patients who conformed to the criteria of possible PSP with symptoms of vertical supranuclear palsy or slowing of vertical saccades, but not prominent postural instability with falls occurring in the first year. Subtypes of PSP were classified according to the reports by Williams et al., (2005, 2007). PSP‐P was clinically diagnosed if the patient demonstrated the clinical features of asymmetric onset, tremor, and a moderate initial therapeutic response to levodopa. For patients with possible PSP, we diagnosed cases with a Parkinson's disease‐like clinical picture with a moderate levodopa response in the first 2 years as clinical PSP‐parkinsonism (cPSP‐P). For possible PSP, we diagnosed cases with gradual onset of gait freezing or speech impairment, an absence of limb rigidity, no dementia, or ophthalmoplegia during the first 5 years, and no sustained response to levodopa as clinical PSP‐pure akinesia with gait freezing (cPSP‐PAGF).

### 2.4 Data analysis

The prevalence day was defined as the date when the number of PSP patients became constant in terms of the change in initial registrations and deaths of the patients. The crude prevalence per 100,000 living people and the 95% confidence interval (CI) were calculated using the population demographics generated from the former Yonago City on the prevalence day of 1 October 2010. The prevalence of each clinical PSP subtype was calculated for the same time. Age‐ and sex‐adjusted prevalence was calculated by direct standardization to the population demographics in Yonago City on the prevalence day of 1 April 1999 and in Japan on the prevalence day of 1 October 2010. In the present study, we used the same clinical diagnostic criteria for PSP that was used in a previous report by Kawashima, et al., (2004). The prevalence of RS (probable RS and possible RS) and PSP (RS, cPSP‐P, and cPSP‐PAGF) were compared between the April 1, 1999 and October 1, 2010 prevalence days.

These studies were approved by the Ethical Review Board of the Tottori University Faculty of Medicine.

## 3 Results

In this investigation, we identified 25 patients with PSP: 16 with probable RS, 4 with possible RS, 3 with cPSP‐P, and 2 with cPSP‐PAGF. During this investigation period, before the prevalence day of 1 October 2010, one patient with probable RS died. After the prevalence day, 11 patients died. Three cases were confirmed pathologically as definite PSP. Table 1 summarizes the characteristics of the three autopsied PSP cases. Case 1 was diagnosed as cPSP‐P, case 2 and case 3 were diagnosed as probable RS. The period from onset to diagnosis of PSP was 3.2 ± 2.5 years. Table 2 summarizes the characteristics of the 25 patients with PSP who were examined in this study.

**Table 1:** Pathologically confirmed patients

	Case 1	Case 2	Case 3
Gender	Male	Female	Male
Age	82	72	77
Age of onset	71	70	76
Symmetry of onset symptoms	Asymmetric	Symmetric	Symmetric
Supranuclear ophthalmoplegia	+	+	+
Postural instability with fall in the first year	–	+	+
Levodopa response in the first 2 years	+	–	–
NINDS‐PSPS criteria	Possible PSP	Probable PSP	Probable PSP
Clinical diagnosis in this survey	cPSP‐P	Probable RS	Probable RS
Pathological diagnosis	Definite PSP	Definite PSP	Definite PSP

RS, Richardson's syndrome; PSP, progressive supranuclear palsy; cPSP‐P, clinical PSP‐parkinsonism.

**Table 2:** Number of patients with RS and PSP subtypes

Subtype	Case (M:F)	Crude prevalence in Yonago City (per 100,000)	Adjusted prevalence in Yonago City in 1999 (per 100,000)	Adjusted prevalence in Japan in 2010 (per 100,000)	AgeMean ± *SD* (year)	Age at onsetMean ± *SD* (year)	Duration of illnessMean ± *SD* (year)	Time to diagnosisMean ± *SD* (year)
RS	20 (7:13)	14.32	10.23	13.80	77.5 ± 7.1	73.6 ± 7.0	4.0 ± 3.5	3.0 ± 2.6
Probable RS	16 (4:12)	11.45	8.07	10.95	78.6 ± 6.7	74.7 ± 6.4	4.0 ± 3.9	2.4 ± 2.6
Possible RS	4 (3:1)	2.86	2.15	2.86	74.5 ± 7.9	69.0 ± 8.2	3.8 ± 1.9	5.3 ± 1.5
cPSP‐P	3 (3:0)	2.15	1.52	2.03	85.0 ± 11.3	76.0 ± 7.1	7.7 ± 5.8	3.5 ± 0.6
cPSP‐PAGF	2 (2:0)	1.43	1.07	1.43	87.5 ± 4.9	76.0 ± 1.4	11.0 ± 7.1	6.0 ± 0.0
Total	25 (12:13)	17.90	11.92	17.26	77.5 ± 6.5	72.5 ± 6.9	5.0 ± 4.4	3.2 ± 2.5

RS, Richardson's syndrome; PSP, progressive supranuclear palsy; cPSP‐P, clinical PSP‐parkinsonism; cPSP‐PAGF, clinical PSP‐pure akinesia with gait freezing.

The crude prevalence of PSP per 100,000 people was 17.90 (male = 18.05; female = 17.76), and the 95% CI was 12.12–26.42 (male = 10.33–31.55; female = 10.38–30.39). The age‐ and sex‐adjusted prevalence of PSP per 100,000 people in Japan on the prevalence day of 1 October 2010 was 17.26 (male = 18.14; female = 16.63), and the 95% CI was 17.03–17.48 (male = 17.81–18.48; female = 16.32–16.95). It was predicted that approximately 22,000 patients suffer from PSP in Japan. The crude prevalence of patients with RS per 100,000 people was 14.32 (male = 10.53; female = 17.76), and the 95% CI was 9.27–22.12 (male = 5.10–21.74; female = 10.38–30.39). The age‐ and sex‐adjusted prevalence of RS per 100,000 people in Japan on the prevalence day of 1 October 2010 was 13.80 (male = 10.62; female = 16.63), and the 95% CI was 13.60–14.01 (male = 10.37–10.88; female = 16.32–16.95).

In this survey, no patients that referred to the University Hospital with a classification of possible PSP were diagnosed with any of the other PSP subtypes, including PSP‐corticobasal syndrome (PSP‐CBS; Williams et al., 2005), PSP‐progressive non‐fluent aphasia (PSP‐PNFA; Williams et al., 2005), or PSP with cerebellar ataxia (PSP‐C; Kanazawa et al., 2009).

## 4 Discussion

**Figure 1:**
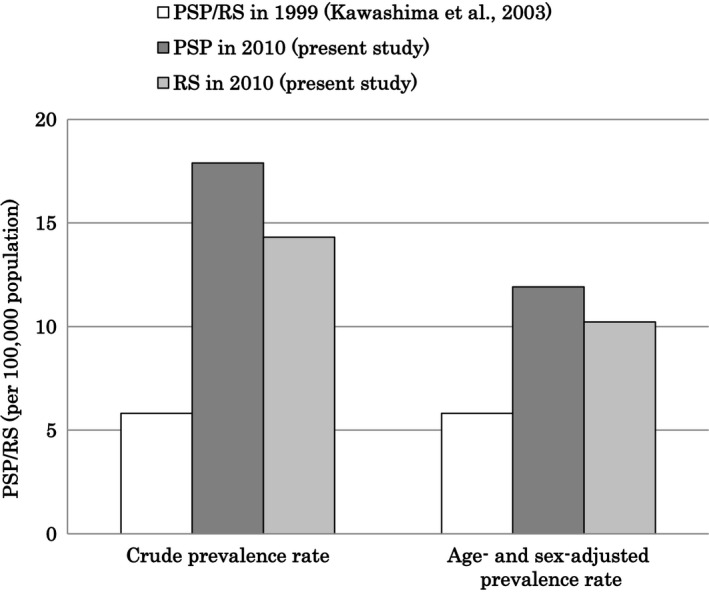
The prevalence of progressive supranuclear (PSP) and Richardson's syndrome (RS). Crude and age‐ and sex‐adjusted prevalences of PSP/RS in 1999, PSP in 2010, and RS in 2010 (adjusted to the population of Yonago City on the prevalence day of 1 April 1999). PSP was conceptually equal to RS in 1999. The subjects in 1999 included only patients with RS according to the diagnostic criteria of the NINDS‐SPSP

This is the first report to investigate the change (during a decade) in the prevalence of PSP using the same surveillance area in Japan. The prevalence of PSP per 100,000 people in Yonago City in 2010 was 17.90 (male = 18.05; female = 17.76). Our present data indicated that the prevalence of PSP per 100,000 people in Yonago City increased compared to 1999 (Kawashima et al., 2004) in which it was 5.82 (male = 9.14; female = 2.75), and the 95% CI was 1.78–9.86 (male = 1.82–16.47; female = −1.08–6.65) (Fig. 1). In the comparison for Yonago City, the age‐ and sex‐adjusted prevalence per 100,000 people in Yonago City on the prevalence day of 1 April 1999 was 11.92 (male = 11.66; female = 13.25), and the 95% CI was 7.38–19.25 (male = 5.83–23.33; female = 7.01–24.76). The number of patients with PSP increased even if adjusted by age and sex. When including only RS, the prevalence of PSP per 100,000 people in Yonago City was greater in 2010 compared to the 1999 study (Fig. 1). The age‐ and sex‐adjusted prevalence per 100,000 people in Yonago City on the prevalence day of 1 April 1999 was 10.23 (male = 6.74; female = 13.25), and the 95% CI was 6.10–17.14 (male = 2.74–16.57; female = 7.09–24.76). The crude and adjusted prevalence of RS per 100,000 people in Yonago City increased compared to 1999. The 95% CI for the adjusted PSP prevalence rate in 1999 overlaps with that in 2010, and the point estimation prevalence in 2010 (17.26 per 100,000) falls within the adjusted 95% CI (7.38–19.25) in 1999. Thus, although the point prevalence estimation increased from 1999 to 2010, we cannot be 95% confident that this estimate is significantly higher than the adjusted estimate for 1999. This problem reflects the small sample size of PSP patients and affects the interpretation of this study.

Several factors may influence the prevalence of PSP. First, an increase in the elderly proportion of the population of Yonago City could contribute to changes in the prevalence of PSP. Second, PSP was certified as part of the Specified Disease Treatment Research Program by the Japanese government in 2003. Patients with PSP were then able to get support for medical services (at the public's expense). Thus, more PSP patients visited hospitals and were diagnosed. Additionally, a nursing‐care insurance system was introduced in 2000 as a social security system corresponding to the aging societies in Japan. These changes in the social security and medical systems in Japan might improve nursing care and lead to an increased recognition and diagnosis of patients with PSP, thereby influencing the prevalence. Third, during the decade since the previous investigation, some PSP subtypes, such as PSP‐P and PSP‐PAGF, were defined. Patients with PSP subtypes presented with the specific pathological PSP findings but with different clinical manifestations. In some cases, it might be difficult to diagnose patients who are in the early phases of PSP subtypes. The long‐term investigation in this study might make it easier to recognize the subtypes. The prevalence of RS in 2010 was higher than the age‐ and sex‐adjusted prevalence of PSP in Yonago City on the prevalence day of 1 April 1999. The overall increase in prevalence was not solely due to the appearance of subtypes, such as PSP‐P and PSP‐PAGF. Although the other subtypes, such as PSP‐CBS (Williams et al., 2005), PSP‐PNFA (Williams et al., 2005), and PSP‐C (Kanazawa et al., 2009), have been reported, no patients were diagnosed with these subtypes in this study.

The prevalence of PSP has also been reported in areas outside of Japan. In several population‐based studies in various countries, reports of PSP prevalence vary from 1.0 to 6.5 per 100,000 people (Chio, Magnani, & Schiffer, 1998; Golbe, Davis, Schoenberg, & Duvoisin, 1988; Nath et al., 2001; Schrag, Ben‐Shlomo, & Quinn, 1999; Wermuth, Joensen, Bunger, & Jeune, 1997), which is in agreement with the results of our previous report: 5.82 per 100,000 people (Kawashima et al., 2004). However, the prevalence of PSP in Kochi, Japan, reported by Osaki, Morita, Kuwahara, Miyano, & Doi (2011) was 18 per 100,000 people, and the age‐ and sex‐adjusted prevalence per 100,000 people in Japan was 10 per 100,000 with a 95% CI of 2–17. These subjects had RS, but the authors did not include patients with PSP‐P or PSP‐PAGF. In that study, the crude prevalence of RS was higher compared to our data. However, when adjusted for age and sex, the adjusted prevalence was lower compared to our data. This indicates that the difference between the two studies may be due to the proportion of the population in the investigated area and the survey method.

We observed almost identical rates for both sexes (12 males and 13 females), which was inconsistent with our previous report (6 male vs. 2 female). The sex‐related difference was not consistent in reports by other investigators. Williams et al. (2005) investigated 103 pathologically confirmed cases of PSP, and 65 (63%) were males. In Santacruz, Uttl, Litvan, and Grafman (1998), female patients with PSP were represented equally (154 males, 164 females) in a sample of living individuals, whereas men formed 61.8% in a sample (73 males, 45 females) of deceased patients. Male patients were diagnosed later after symptom onset than female patients (males, 33.4 months; females, 24.1 months) and had more severe symptoms and a shorter duration from diagnosis to death (males, 37.0 months; females, 47.6 months). In contrast, dell'Aquila et al. (2013) reported no differences regarding time to diagnosis, disease duration, or survival between males and females in a clinical investigation of 43 PSP patients (53.5% males). The results of previous reports are therefore discrepant. The population of females increased relative to the change in the elderly proportion in Yonago City, and the time to diagnosis for male patients was longer than for female patients. The sex difference in patients with PSP thus remains unclear.

There were some limitations to this study. Most patients received clinical diagnoses of PSP, and only three cases were confirmed pathologically. The subjects were recruited according to the NINDS‐PSPS criteria, and possible RS cases were classified as a clinical subtype. The cases that failed to meet the NINDS‐PSPS diagnosis criteria were eliminated in this survey. Clinical diagnoses of PSP‐P or PSP‐PAGF were not allowed due to the lack of clinical criteria, therefore in this study we provide new criteria for cPSP‐P and cPAGE. Further investigation will be required to determine the accuracy of these clinical criteria using confirmed pathological cases. In this study, the participants were diagnosed via detailed neurological examinations by multiple neurological specialists at the University Hospital and were evaluated via magnetic resonance imaging of the brain and by 123‐I‐meta‐iodobenzylguanidine myocardial scintigraphy, which effectively differentiates PSP from Parkinson's disease (Braune, Reinhardt, Schnitzer, Riedel, & Lucking, 1999; Orimo, Ozawa, Nakade, Sugimoto, & Mizusawa, 1999; Rascol & Schelosky, 2009). Therefore, the diagnostic accuracy was high. This study was an investigation conducted through medical institutions, and some patients with PSP were not able to be contacted for this survey. Given that some patients were not referred to the University Hospital from the general hospital, it is possible that referral bias might exist in this study. Moreover, some patients that underwent medical treatment at home may not have been included in this investigation because the design of this investigation was not a door‐to‐door study. Epidemiologic problems, such as immigration to other cities or being diagnosed at other hospitals out of this region, are other factors that may have excluded some patients. Patients with PSP may be admitted to nursing homes without visiting neurologists or with a misdiagnosis because PSP is insidious in onset and progresses slowly. PSP is currently difficult to diagnose. Most patients who were diagnosed with rare neurodegenerative diseases, such as PSP, visited the University Hospital for diagnosis more than one during their clinical course around the Yonago area. In this study, the period from onset to diagnosis of PSP was approximately 3 years, whereas this investigation was conducted over 6 years. Investigating for a long period prevented PSP patients from dropping out of this study. We believe that our data include most of the patients with PSP in the investigational area.

In conclusion, the prevalence of PSP in Yonago City, Japan, increased during the 11‐year period from 1999 to 2010. The increased prevalence of PSP in 2010 might be due to several factors, including the aging population. The adjusted prevalence of RS per 100,000 people in 2010 was still greater than the prevalence of PSP per 100,000 people in 1999. The increasing prevalence of PSP may be due not only to population aging but also to the inclusion of other phenotypes or the improved awareness of the condition. PSP was certified by the Specified Disease Treatment Research Program of the Japanese government in 2003, which subsidizes medical care for patients with rare and intractable diseases. An increase in PSP diagnoses may be due to the improvement in social systems, allowing for better patient identification.

## Conflicts of Interest

The authors declare no conflicts of interest.
